# Determination of Heavy Metal Concentrations in Normal and Pathological Human Endometrial Biopsies and *In Vitro* Regulation of Gene Expression by Metals in the Ishikawa and Hec-1b Endometrial Cell Line

**DOI:** 10.1371/journal.pone.0142590

**Published:** 2015-11-23

**Authors:** Erwan Guyot, Yevgeniya Solovyova, Céline Tomkiewicz, Alix Leblanc, Stéphane Pierre, Souleiman El Balkhi, Marie-Aude Le Frère-Belda, Fabrice Lecuru, Joël Poupon, Robert Barouki, Martine Aggerbeck, Xavier Coumoul

**Affiliations:** 1 INSERM UMR-S 1124, Toxicologie Pharmacologie et Signalisation Cellulaire, 45 rue des Saints Pères, 75006 Paris, France; 2 Laboratoire de toxicologie biologique, AP-HP, Hôpital Lariboisière, 2, rue Ambroise Paré, 75475 Paris, France; 3 Service de chirurgie gynécologique et cancérologique, hôpital européen Georges-Pompidou, AP-HP, 20, rue Leblanc, 75015 Paris, France; 4 Université Paris Descartes, Paris Sorbonne Cité, Centre universitaire des Saints-Pères, 45 rue des Saints Pères, 75006 Paris, France; 5 AP-HP, Hôpital Necker-Enfants Malades, service de biochimie métabolique, 149, rue de Sèvres, 75743 Paris, France; 6 Institute of Urgent and Recovery Surgery named after V.K. Gusak of the Ukrainian Academy of Medical Sciences, Donetsk; 47, avenue Leninsky, Donetsk- 83045, Ukraine; University of Quebec at Trois-Rivieres, CANADA

## Abstract

It is well known that several metals, such as lead, mercury, cadmium, and vanadium, can mimic the effects of estrogens (metallo-estrogens). Nevertheless, there are only a few studies that have assessed the effects of toxic metals on the female genital tract and, in particular, endometrial tissue. In this context, we measured the concentrations of several trace elements in human endometrial tissue samples from individuals with hyperplasia or adenocarcinoma and in normal tissues. Hyperplasic endometrial tissue has a 4-fold higher concentration of mercury than normal tissue. Mercury can affect both the AhR and ROS signaling pathways. Thus, we investigated the possible toxic effects of mercury by *in vitro* studies. We found that mercury increases oxidative stress (increased HO1 and NQO1 mRNA levels) and alters the cytoskeleton in the human endometrial Ishikawa cell line and to a lesser extent, in the “less-differentiated” human endometrial Hec-1b cells. The results might help to explain a potential link between this metal and the occurrence of endometrial hyperplasia.

## Introduction

The term “heavy metals” is used often for the most widespread toxic metals, among which are lead, cadmium and mercury. Many of them are found both naturally and as a result of multiple human activities. These metals are highly toxic and cause numerous symptoms and pathologies, such as neurological effects for mercury [1]. Furthermore, one of the main characteristics shared by these metals is their environmental persistence [2]. Environmental contamination thus remains an important health issue even if efforts have been undertaken to reduce the use of these metals. Depending upon the metal and its uses, human exposure may vary. The central nervous system is the main target organ that has been identified for the deleterious effects of toxic metals [3–6]. Little data exist concerning the accumulation of metals in other organs, including the reproductive organs, particularly in women, despite the fact that several heavy metals are described as endocrine disruptors [7].

The endometrium is the internal part of the uterus composed of both epithelial and stromal components. This tissue undergoes major modifications during the menstrual cycle and is highly sensitive to estrogens and progesterone. The first evidence for the accumulation of trace elements in the endometrium was published in the 1970s. Using neutron activation analysis, 31 samples were evaluated for 25 elements and significant cyclic variations for some of them, including cadmium, were found. Nevertheless, this method is not suitable to detect some elements due to poor sensitivity of the assay [8].

“Heavy metals” may activate multiple signaling pathways, including those regulated by nuclear receptors, the transcription factors Nrf2 and the Aryl hydrocarbon Receptor (AhR). In addition to their effects on signaling pathways, heavy metals also induce oxidative stress. Reactive oxygen species (ROS) generated by exposure to metals such as cadmium, have been linked to deleterious effects [9]. The effects of chronic exposure to metals are more difficult to assess and few studies have tackled the possible effects of metals on reproductive organs.

In this study, we measured the concentrations of different metals in human uterine endometria under different pathological conditions. We report, for the first time, an increased content of mercury in hyperplastic endometrial tissue as compared to normal tissue. Based on these results, we performed an *in vitro* study on the effects of heavy metals in the Ishikawa human endometrial cell line, focusing on mercury. We found that this metal induces several markers of oxidative stress accompanied by a decrease of cell adhesion markers as shown by a decrease in the expression of paxillin at focal adhesion sites and a loss of actin stress fibers.

## Material and Methods

All experiments have been approved by the INSERM UMR-S 1124 Institutional Advisory Board. A complete version of the Material and Methods can be found in the S1 File.

### Clinical studies

#### Tissue samples, measurements of heavy metals and statistical analysis

Human paraffin embedded samples were obtained from patients with different endometrial pathologies: typical endometrial hyperplasia, endometrial cancer and normal endometrial tissues. Atypical hyperplasias were excluded. Control samples were obtained from patients not having an identified endometrial pathology. All patients were pre-menopausal women less than 50 years old. According to articles L. 1122-1-1 & L. 1211–2 (Code de la santé publique, France; Code of Public Health), the secondary use for medical or scientific purposes of elements or products of the human body collected for other purposes is permissible if the individuals from whom the material has been collected have been informed of the secondary use, and he/she (or his/her representative) has not objected to such use. However, the obligation to inform the individuals can be waived if it is impossible to find the person (practical impossibility to re-contact). This is the case in our study in which the samples were collected from 2002 and 2009 after obligatory surgery and conserved as wastes for further research. As a consequence, the institutional advisory board waived the need for consent.

All samples were dried and digested to obtain solutions containing 1g of dry tissue per liter. Metal-free control samples were also prepared and analyzed for each digestion series. The solutions were directly analyzed by inductively-coupled plasma mass spectroscopy (ICP-MS, see **S1 Table: ICP-MS parameters).**

The data are expressed as the mean ± SEM (standard error of the mean). Differences between groups were analyzed by Student two-tailed t-tests. Nemenyi’s test was used for the quantification of metals in biopsies. A p-value < 0.05 was considered to be statistically significant (*** p<0.001;** p<0.01; * p<0.05).

### Cell studies

#### Cell culture and chemical products

The Ishikawa and Hec-1b cell lines are derived from an endometrial adenocarcinoma and were purchased respectively from Sigma-Aldrich^TM^ (catalogue number 99040201) and ATCC (catalogue number HTB-113). Cell culture conditions are described in S1 File. Metal salts (VoSO_4_ 5H_2_O), (HgCl_2_), (Pb(NO_3_)_2_), (CdCl_2_) were purchased from Sigma-Aldrich^TM^. Each metal salt was dissolved in water and sterile-filtered before cell treatment. Cells were incubated with various concentrations of metals or N-Acetyl Cysteine (10 mM, Sigma) or TCDD (25 nM, LCG Promochem) for 24h or 48h.The “CellTiter 96® Aqueous cell proliferation assay” (Promega^TM^, France) was used to determine the number of cells. For cell counting, Ishikawa cells were plated into 12 well plates (100,000 cells per well) and treated with 3μM of HgCl_2_. Forty-eight hours later, the cells were counted using the same proliferation assay. For immunofluorescence studies, cells were seeded onto glass coverslips at a density of approximately 5x10^5^ cells per well in 6-well plates. Cells were treated with 3μM HgCl_2_ for 48 h in DMEM without phenol red and supplemented with 3% charcoal-stripped fetal calf serum (see details in the S1 File).

#### RNA preparation and quantitative Reverse Transcription PCR

For most experiments, 0.2 million cells were seeded into 6-well plates and treated, or not, 2 days later, with the compounds indicated in the Figures. RNA was prepared using the RNeasy mini kit from Qiagen (France). Reverse transcription was performed using the High Capacity cDNA Reverse Transcription Kit (Applied Biosystems, France) prior to quantitative PCR performed with 40 ng of cDNA [10], with duplicates for each experiment.

#### xCELLigence real-time cell analysis

Endometrial cells were seeded in duplicate for each condition into 16-well xCELLigence E-plates at 10,000 cells/well in a final volume of 160 μL and immediately treated or not with 3 or 10μM HgCl_2_. The impedance value of each well was automatically monitored by the xCELLigence system and expressed as a cell index value (CI). Initial attachment and spreading were monitored by measuring the CI every 15 minutes for the first twenty hours. The rate of attachment (CI/h) was calculated using the xCELLigence RTCA software (ACEA Biosciences, Inc., Roche Diagnostics). Each experiment was performed in triplicates. For migration studies, Ishikawa cells (50,000 cells per well) were seeded into CIM-16 plates (which are specifically used to measure cell migration) in DMEM without phenol red supplemented with 3% charcoal-treated (desteroidized) FBS. The lower chambers contained media with 10% FBS in order to assess chemotactic migration. After 24 h of incubation at 37°C, the medium was replaced and the cells were treated, or not, with 3 or 10 μM of HgCl_2_. The impedance value of each well was automatically monitored by the xCELLigence system and expressed as a cell index value (CI). Higher CI values equate to more migration; the cells migrate from the upper chamber towards the lower chamber. The migration curves were monitored online, every 5 min during the incubation and the slope of the migration curve was calculated using the RTCA 1.2 software. Each experiment was performed in triplicates (n = 4).

### Statistical analysis

The values are expressed as mean ± SD. The data were analyzed by analysis of variance (ANOVA) followed by Fisher's Least Significant Difference test to examine the differences between the different groups. A value of p < 0.05 was considered to be statistically significant.

## Results

### Measurement of metal concentrations in the endometrial tissues

**Fig 1:**
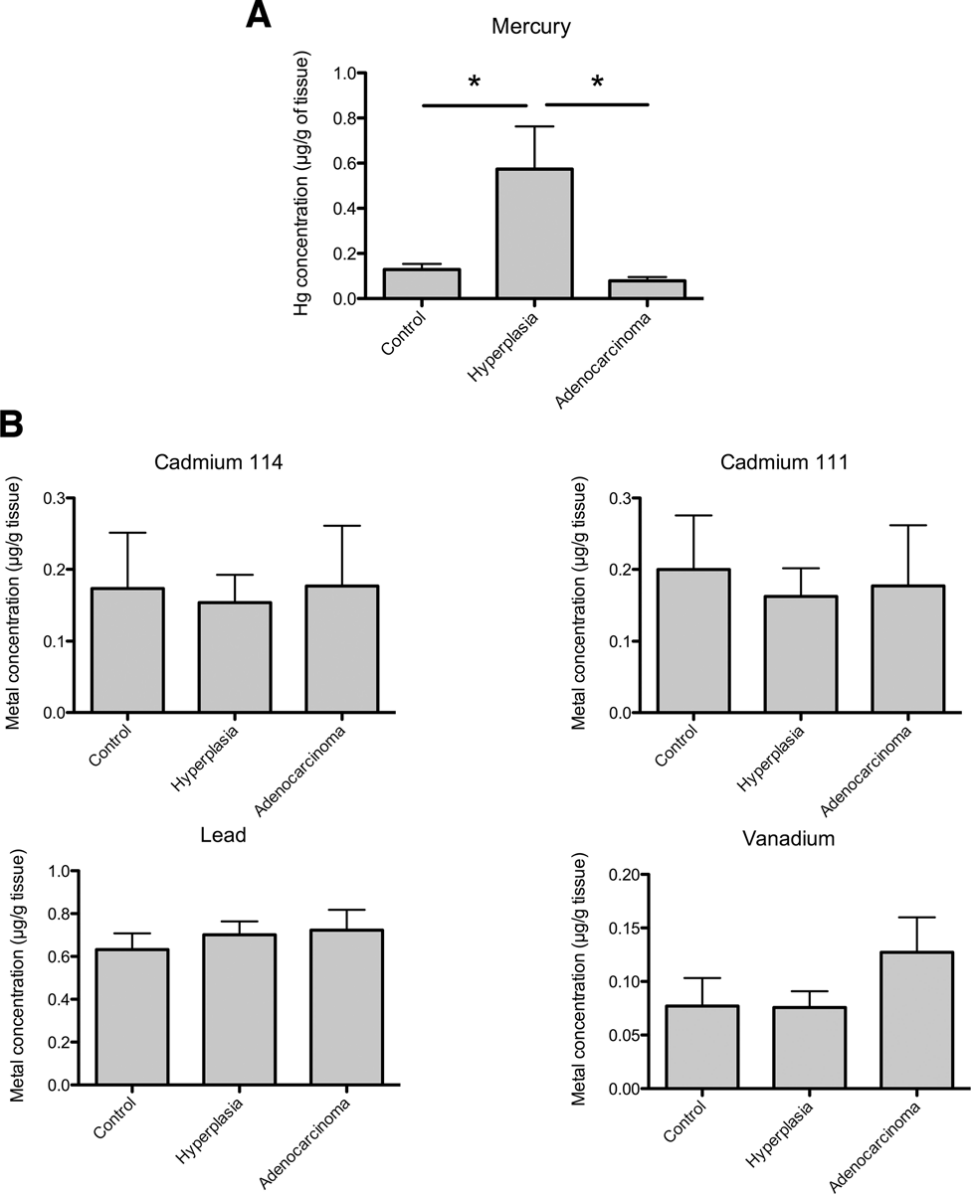
Measurement of the mercury (1A) and of the lead, cadmium 111, cadmium 114 and vanadium (1B) content in endometrial tissues with different pathophysiological characteristics (normal tissues (control), hyperplasia, adenocarcinomas). The results are expressed as μg of metals/g of wet tissue. Nemenyi’s test has been used to determine statistical significance with * p< 0.05 (mercury). No statistical difference has been determined for the other metals.

The concentrations of mercury, lead, cadmium (isotopes 111 and 114) and vanadium were determined in 14 normal, 10 hyperplastic and 11 adenocarcinoma biopsies by ICP-MS. The concentrations of all the metals were measurable, exhibiting non-zero levels. Among them, there was a significantly higher level of mercury in the hyperplasia tissues as compared to the control tissues (Fig 1A) whereas no difference was observed between the control and the adenocarcinoma tissues (Fig 1A). No statistically significant difference was observed for the other metals tested (cadmium, lead, vanadium) (Fig 1B). However, there was a large variability in the levels of both Cd 111 and Cd 114 in the hyperplasia tissues and in some individuals, very high levels were found.

### Measurement of the Ishikawa endometrial cell viability upon metal treatment

The effects of the heavy metals were investigated in an endometrial cell line. Endometrial hyperplasia is a pre-tumor state; while less differentiated than normal endometrial cells, hyperplasic cells remain in a differentiated state. We then chose the well-differentiated adenocarcinoma Ishikawa cells to perform our studies. The cell viability was measured to determine the range of concentrations that did not generate cell mortality. The concentration at which cell viability decreased varies greatly with the metal tested (S1 Fig). Cadmium was the most toxic with 70% mortality at 10μM. Vanadium and mercury (both 20 μM) reduced the viability of the cells by 60% and 35%, respectively, whereas concentrations > 500 μM were required to decrease cell viability for lead.

### Effects of mercury and TCDD on gene markers for AhR activation and oxidative stress in Ishikawa cells

Since mercury was the only metal for which there was a significant difference in concentration between control and pathological tissue samples, we studied its effects on gene expression in Ishikawa cells. Mercury affects both the Aryl hydrocarbon Receptor (AhR) and ROS signaling pathways (Amara and El-Kadi, 2011). The levels of mRNA expression of two genes altered by the activation of the AhR (CYP 1A1 and CYP 1B1) and two markers of oxidative stress (Heme oxygenase 1, HO1 and NADP Quinone Oxydoreductase 1, NQO1, which also reflects AhR activation) were measured in Ishikawa cells treated for 24 or 48 hours with various concentrations of HgCl_2_ (S2 and S3 Tables, respectively). There was no increase in the level of CYP1A1 mRNA (except at 20 μM where cell viability is decreased by 35%), CYP1B1 and AhR mRNA. However, mercury significantly increased, in a dose-dependent manner, the quantity of HO1 mRNA from 0.3 μM to 20 μM. The level of NQO1 mRNA was also increased, albeit at a lower level.

**Fig 2:**
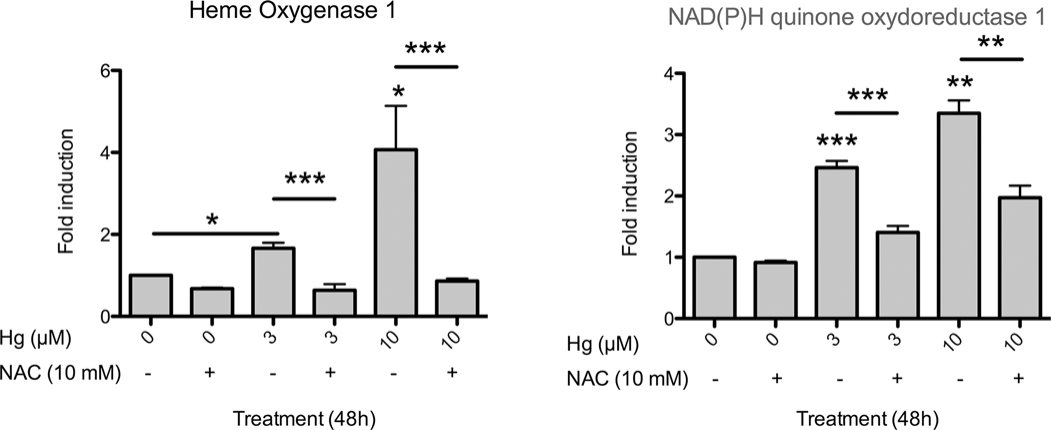
Relative mRNA levels of HO1 and NQO1 in Ishikawa cells exposed or not for 48h to 3 or 10 μM HgCl_2_ or to 10 mM of N-AcetylCysteine (NAC) alone or in combination with mercury. Quantitative RT-PCR was used in this experiment. The results, from five independent experiments, are expressed as the mean ± SD (standard error of the mean). Differences between groups were analyzed by Student two-tailed t-tests. A p-value < 0.05 was considered as statistically significant (*** p<0.001, ** p<0.01; * p<0.05).

The increase in the two markers of oxidative stress in Ishikawa cells at non-toxic concentrations suggests that mercury triggers oxidative stress in these cells. In contrast, mercury had no effect on the expression of AhR or two well-known AhR target genes, CYP1A1 and CYP1B1. To determine whether ROS were responsible for the increase in the expression of HO1 and NQO1, we co-treated the cells with mercury and an anti-oxidative compound, N-acetylcysteine (NAC). NAC, a precursor of cysteine and of glutathione, counteracted totally (HO1) or partially (NQO1) the effects of mercury on the expression of both genes (Fig 2), which suggests that the increase in the expression of these genes is related to an oxidative stress triggered by mercury.

We also assessed the effects of the other metals (cadmium, lead and vanadium) detected in the endometrial tissues, on the mRNA levels of the CYP1A1, CYP1B1, HO1, NQO1 and AhR genes in Ishikawa cells. Cadmium was the most potent inducer of HO1 (21- and 13-fold increases in mRNA levels at 24 and 48h, respectively, were observed) probably reflecting effects on oxidative stress (S4 Table). Similar inductions of HO1 and NQO1 were observed for mercury (10 μM) and lead (300 μM). Cadmium was the only metal that gave a detectable and statistically significant effect (although small) at the chosen time points on CYP1A1 expression (about a 2-fold increase). Vanadium (3 μM) did not affect the expression of any of the selected genes.

Although HgCl_2_ had no significant effect on the expression either CYP1A1 or CYP1B1 at non-toxic concentrations, we hypothesized that mercury might influence the activity of the AhR, as it was reported previously [11,12]. Interestingly, mercury and dioxins are sometimes described as food co-contaminants, particularly in fish [13]. We treated Ishikawa cells with mercury and a prototypical ligand of the AhR, TCDD (2,3,7,8 TetraChloroDibenzo-*para*-Dioxin). We first confirmed that treatment with 25 nM TCDD (48h) alone did not alter the viability of the cells (data not shown). Further, TCDD in combination with mercury (48h) did not decrease cell viability to a greater extent than mercury alone (data not shown). Then, the mRNA levels of the HO1, NQO1, CYP1A1 and CYP1B1 genes were measured in cells treated with either 25nM TCDD or 10μM HgCl_2_ or their combination for 24 or 48h. Treatment with TCDD alone (time) did not have a statistically significant effect on the expression of both HO1 and NQO1 (the 1.5-fold increase was not statistically different from the control at this time point) and had the expected effect on the 2 CYPs tested (S4 Table). The effect of the combination of TCDD and HgCl_2_ on the expression of CYP1A1, CYP1B1, HO1 and NQO1, also was not statistically different from the effect of the mercury salt or TCDD (S5 Table).

### Effects of mercury on cell phenotype in Ishikawa cells

**Fig 3:**
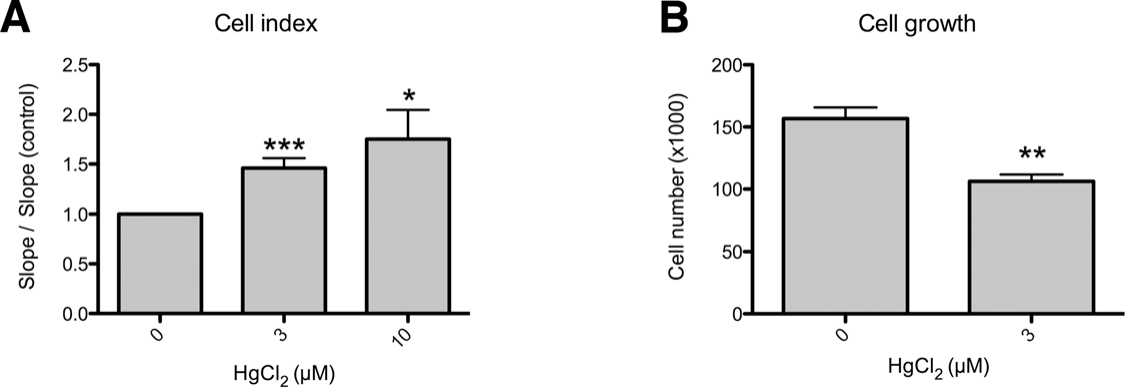
Mercury alters the cell index and proliferation of Ishikawa cells. (A) Relative rate of change of the cell index of Ishikawa cells exposed or not for 48h to 3 or 10 μM HgCl_2_. The cell index (CI) profiles of HgCl_2_ 0, 3 and 10 μM-treated cells reflect the response to the respective treatments during the initial cell attachment and the logarithmic growth phase. CI values were recorded every 15 minutes using the RTCA DP System. The results are the mean ± SD (duplicate) and are representative for five different experiments (n = 5). The slope (which represents the rate of change of the cell index) was calculated for the period 5–20h for each treatment. (B) Cell growth was estimated 48h after treatment by malassez cell counting. Each graph represents means ± SEM of 8 measurements. A p-value < 0.05 was considered as statistically significant (*** p<0.001, ** p<0.01; * p<0.05).

**Fig 4:**
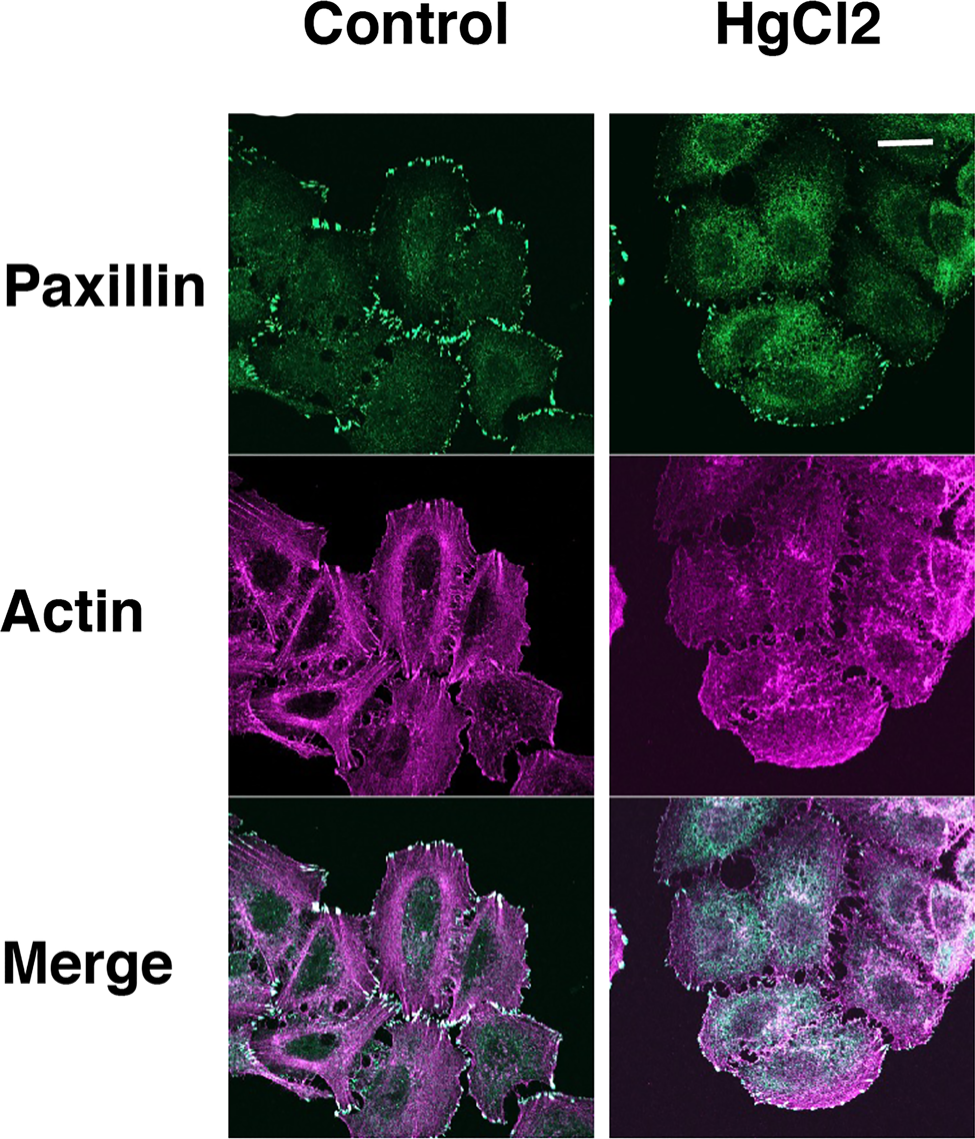
Mercury provokes early morphological changes and redistribution of focal adhesion sites in Ishikawa cells. Ishikawa cells were treated or not for 48h with 3 μM HgCl_2_ and stained with phalloidin for actin (purple, middle part of the panel) and paxillin antibody (green, upper part of the panel). Scale bar 10 μm. The merged images are shown in the lower panel.

As shown previously, cell viability was not modified by concentrations of HgCl_2_ lower than 10 μM (S1 Fig). In order to investigate further a possible link between mercury exposure and endometrial hyperplasia, we studied the effects of the metal on the morphology of Ishikawa cells, using the xCELLigence system. The xCELLigence system is used to non-invasively monitor in real-time conditions, the proliferation, the morphology or the adhesion of cultured cells using electrical impedance as the readout. The continuous monitoring of the impedance is expressed as a cell index value (CI). Treatment with 3 μM HgCl_2_ led to a significant increase in the slope which could reflect increased cell proliferation or adhesion and/or modifications of cell morphology (Fig 3A). We then determined by cell counting, that treatment with 3 μM HgCl_2_ decreases the cell number as compared to control conditions (Fig 3B). Therefore, the increase of CI is not due to an increase in cell proliferation. Morphological changes induced by HgCl_2_, were then evaluated by examining the actin cytoskeleton and paxillin, a marker of focal adhesion sites. In the absence of mercury, Ishikawa cells displayed an epithelial cell-like morphology (Fig 4). Actin fibers were arranged longitudinally and paxillin staining was observed at the cell periphery. In contrast, cells exposed for 48h to 3 μM HgCl_2_ exhibited a reduced network of actin, a possible loss of stress fibers and a decreased expression of paxillin at the cell membrane (relocated into the cytoplasm). These morphological changes characterized by cell spreading are consistent with the observed increase in cell index after HgCl_2_ treatment.

We next assessed if the effects of mercury on cell morphology were not due to an epithelial-mesenchymal transition (EMT). We measured the mRNA levels of the E-Cadherin, Vimentin, Snail and Slug in mercury-treated Ishikawa cells. We did not observe any significant change, suggesting that the morphological changes are not due to an EMT (S2A Fig). Moreover, measurement of cell migration by the XCelligence system shows that mercury inhibits cell migration at 10μM (S2B Fig). Then, we concluded that mercury dramatically induced morphological (cell spreading) and cytoskeleton (actin, stress fibers, paxillin) changes but not as part of an EMT program.

### Comparison of the effects of mercury on two endometrial cell lines

We next decided to compare the effects of mercury between the “well-differentiated” adenocarcinoma Ishikawa cells and the “less-differentiated” Hec-1B cells (which do not express for example, estrogen receptor α and progesterone receptor). We then measured the mRNA levels of the CYP1A1, HO1, NQO1, AhR, E-Cadherin, Vimentin and Slug in mercury-treated Hec-1B cells (S3 and S4 Figs). We showed that mercury did not alter significantly the expression of most of the genes tested except at the 10 μM concentration of HgCl_2_ (expression of HO1, E-cadherin and Vimentin). These results suggest that some effects of mercury are common for both cell lines (ex: increased expression of HO1) but may also be different.

## Discussion

Metals are among the most worrisome environmental contaminants and they have been linked to several health issues. Most studies on the effect of toxic heavy metals have been focused on few organs or pathologies, such as the impact on neurological functions or their role in carcinogenicity. Surprisingly the impact of heavy metals on the female reproductive system has been less studied, despite the critical impact of hormones on this system and the finding that many pollutants are endocrine disruptors [7,14]. In MCF-7 cells, cadmium stimulates cell proliferation and activates estrogen receptors (ER) [15]. Ovariectomized rats exposed to cadmium exhibit an increase of uterine volume along with the induction of estrogen-regulated genes [16]. Thus, cadmium is considered to be an estrogeno-mimetic compound. Other metals (lead, zinc and mercury) trigger endometrial changes that promote spontaneous abortions in albino Wistar rats or exhibit an estrogen-like effect in uterotrophic assays [17]. The estrogen-like effect of mercury is blocked by the estrogenic antagonist ICI182780 but no competition for binding to the estrogen receptor between estradiol and mercury was observed [18]. Thus, *in vitro* and *in vivo* studies have shown that some metals are able to modify endometrial homeostasis and to stimulate uterine epithelial cell growth.

Our study is, to our knowledge, the first to measure the amounts of heavy metals in different endometrial biopsies (normal or pathological). We focused on endometrial hyperplasia and cancer for which proliferation depends upon exogenous pro-estrogenic stimuli. Our results showed a significant, 4-fold increase in the level of mercury in hyperplastic tissues. We did not find significant differences for the other metals tested (lead, cadmium and vanadium); however, there was a large variability in the amounts of Cd in hyperplastic tissues and some individuals displayed high levels of this metal. Interestingly, the level of mercury is not significantly different between normal and cancerous tissues. The latter observation may seem surprising in light of the increased levels of mercury in hyperplasia. However, it is possible that cancer cells acquire specific features, which allow them to eliminate metals such as mercury as part of their adaptation to oxidative and xenobiotic stress. For example, cancer cells might over-express xenobiotic transporters, which are commonly described as proteins involved in chemotherapy resistance [19].

Considering the high level of mercury in hyperplasia, we focused on this metal and studied its effects in a human endometrial cell line (Ishikawa). The expression of two well-known gene markers of oxidative stress, HO1 (Heme Oxygenase) and NQO1 (NAD(P)H quinone oxydoreductase 1), was increased when cells were treated with mercury, suggesting the production of reactive oxygen species (ROS). Mercury has been shown to deplete thiol containing anti-oxidants/enzymes and glutathione, which leads to an increase in the levels of superoxide anion radicals, hydrogen peroxide, and hydroxyl radicals [20,21]. As expected, lead and cadmium, two other metals that induce oxidative stress were also able to increase the expression of the same target genes. The involvement of oxidative stress in mercury-mediated induction of HO1 and NQO1 is supported by the effects of NAC, an anti-oxidant compound, which blocked the effect of mercury. To our knowledge, our study is the first one to explore the effect of metal salts on the oxidative stress response in an endometrial lineage.

ROS signaling triggers proliferation in several cellular models, which suggests that this signaling might be the link between chronic mercury exposure and endometrial hyperplasia. This is in line with *in vivo* and epidemiological studies, which have linked exposure to mercury to modifications of reproductive organ physiology. Indeed, the inhalation of mercury vapors disturbs the estrous cycle in rats [22]. Moreover, in humans, menstrual-cycle abnormalities such as painful menstruation and changes in bleeding patterns and cycle durations have been observed in female workers in various occupations associated with exposure to mercury vapor. Furthermore, a study of female dental assistants demonstrated a decrease in fertility in the women most exposed to mercury [23]. In addition to the production of ROS, *in vitro* experiments have suggested other mechanisms that may explain the link between exposure to mercury and some cellular effects. Analyses of gene expression have shown that mercury can be considered as a weak estrogen in the breast-derived cell-line MCF7 [7,24]. Mercury (among other metals) was found to modulate the expression of phase II xenobiotic metabolizing enzymes NQO1 and GSTA (Glutathione S-Transferase A) in a time and dose-dependent manner [25,26]. Considering these results and the fact that the Aryl hydrocarbon Receptor (AhR) is known to regulate the expression of several xenobiotic-metabolizing enzymes, we hypothesized that the AhR might be involved in the effects of mercury in our experiments within the Ishikawa cell line. This hypothesis is of interest considering the reported environmental co-contamination by mercury and AhR ligands. Therefore, we evaluated the effect of mercury on the regulation of well-studied AhR target genes (CYP1A1 and CYP1B1) in the Ishikawa cell line. The regulation of CYP1A1 by metals (including mercury) has been investigated previously in other cell lines [27]. Mercury can act at different levels on CYP1A1 regulation and its effects depend upon the cell line used for the study. Also, opposite effects may be observed depending on the parameter studied [12,28]. The increased level of both CYP1A1 and CYP1B1 mRNA by TCDD suggests that the AhR signaling pathway is functional in the Ishikawa cell line (S4 Table) but our results indicate that there is no significant regulation by mercury of these AhR target genes.

We next investigated the effects of mercury on the cell phenotype. Using the XCelligence technology, we found that mercury modifies Ishikawa cell morphology. The appearance of hyperplastic tissue in the endometrium might be due to an increased cell proliferation but also to an alteration of cell density related to modifications of cell morphology, adhesion or volume. Although we observed a decrease in cell proliferation of Ishikawa cells upon exposure to mercury, there was a clear alteration of the cell phenotype not associated to an epithelial mesenchymal transition. This is consistent with other studies on alternative models, which have shown that mercury impacts the expression of cytoskeletal proteins and cell volume [29–31]. In some of these studies, the mechanisms implicated concerned the formation of cytoplasmic blebs and activation of Ca^2+^-dependent calpain. In addition, the disappearance of actin fibers was also observed in mercury-treated Ishikawa cells. Mercury interacts with tubulin and blocks assembly of microtubules [32–34]. These effects occur mostly at concentrations higher than 10μM whereas we noted the disappearance of stress fibers at 3 μM in Ishikawa cells. Interestingly, perturbations of cell adhesion to the extracellular matrix are observed in a variety of endometrial disorders, including endometrial hyperplasia and cancer [9,35–37]. Considering that endometrial hyperplasia is defined by both increased cellular proliferation and alteration of cell phenotype as a pre-tumoral state, we propose that mercury only contributes to the alteration of cell phenotype and that other factors are involved in stimulation of cell proliferation. As previously discussed, other metals or potent endocrine disruptors display estrogen-like effects and could be responsible for cell proliferation. Finally, it is also interesting to note that mercury effects on endometrial cells is certainly dependent upon the differentiation state. Indeed, using the less differentiated Hec-1B cell line, we did not observe similar results than with the Ishikawa cells. The expression of nuclear receptors is different between both cell lines; for example, Hec-1B cells do not express progesterone receptor and estrogen receptor α. As suggested above, mercury may act through activation of estrogen receptor and display an estrogen-like effect. Then, it would be interesting to test the contribution of the estrogen-signaling pathway to our results in the Ishikawa cell line. Moreover, it has been shown that progesterone is a major endometrial tumor suppressor [38] which inhibits the invasiveness of endometrial cells [39]; HEC-1B cells do not express the progesterone receptor and we could hypothesize that binding of mercury on this protein could also partly explain the differences we observed between both cell lines. Overall, this suggests that the differentiation state of the cell line is an important parameter to consider in those studies, reinforce our choice to initially use a “well-differentiated” cell line matching the properties of hyperplasic cells and open new perspectives to understand the mechanisms triggered by mercury comparing “well- and less-differentiated” endometrial cell lines.

In conclusion, our study shows that the amount of mercury found in endometrial hyperplastic tissue is higher than in control endometrium and in endometrial cancers. *In vitro* studies indicate that mercury increases ROS and some, but not all, the hallmarks of cancer since significant morphological changes but not proliferation were induced. This suggests that mercury could contribute at a specific stage to endometrial dysplasia but is not capable alone to induce the full-blown cellular proliferation characteristic of cancer. We speculate that pollutant mixtures including mercury and other chemicals with proliferative action could constitute potent endometrial cancer promoters. A larger study with more extensive epidemiological data is clearly needed to confirm our preliminary results and could better highlight the real impact of mercury along with other substances in endometrium pathologies.

## Supporting Information

S1 Fig Viability of Ishikawa cells after 48h of treatment with various metals. Ishikawa cells were treated with the various metals for 48h and the viability measured with the “CellTiter 96® Aqueous cell proliferation assay”. The results are expressed as the percentage of viability for untreated cells (100%) (n = 3).
(TIF)

S2 Fig **A. Relative mRNA levels of E-Cadherin, Vimentin, Snail and Slug in Ishikawa cells exposed or not for 48h to 3 or 10 μM HgCl_2_ or to 5 mM of N-AcetylCysteine (NAC) alone or in combination with mercury**. Quantitative RT-PCR was used in this experiment. The results, from five independent experiments, are expressed as the mean ± SD (standard error of the mean). Differences between groups were analyzed by Student two-tailed t-tests. **B. Effect of mercury on the rate of migration of Ishikawa cells.** Ishikawa cells were incubated in the CIM -plate and treated with 3 or 10μM of mercury. The rate of migration was monitored in real-time using the xCELLigence system (*n* = 4). The results are expressed as measurements of the CI (***, *P*<0.001, *n* = 4).
(TIF)

S3 Fig Relative mRNA levels of AhR, CYP1A1, HO1, NQO1 in HEC-1B cells exposed or not for 48h to 3 or 10 μM HgCl_2_ or to 5 mM of N-AcetylCysteine (NAC) alone or in combination with mercury. Quantitative RT-PCR was used in this experiment. The results, from five independent experiments, are expressed as the mean ± SD (standard error of the mean). Differences between groups were analyzed by Student two-tailed t-tests (***, *P*<0.001).
(TIF)

S4 Fig Relative mRNA levels of E-Cadherin, Vimentin and Slug in HEC-1B cells exposed or not for 48h to 3 or 10 μM HgCl_2_ or to 5 mM of N-AcetylCysteine (NAC) alone or in combination with mercury. Quantitative RT-PCR was used in this experiment. The results, from five independent experiments, are expressed as the mean ± SD (standard error of the mean). Differences between groups were analyzed by Student two-tailed t-tests (***, *P*<0.001).
(TIF)

S1 File A complete version of the “Supplementary Material and Methods” section. (DOCX)

S1 Table ICP-MS parameters. (DOCX)

S2 Table Relative levels of HO1, NQO1, CYP1A1, CYP1B1 and AhR mRNA in Ishikawa cells exposed to different mercury concentrations for 24h measured by quantitative RT-PCR. A p-value < 0.05 was considered as statistically significant (*** p<0.001; ** p<0.01; * p<0.05) (n = 3).
(DOCX)

S3 Table Relative levels of HO1, NQO1, CYP1A1, CYP1B1 and AhR mRNAs in Ishikawa cells exposed to different mercury concentrations for 48h measured by quantitative RT-PCR. A p-value < 0.05 was considered as statistically significant (*** p<0.001; ** p<0.01; * p<0.05). UD: undetermined (n = 3).
(DOCX)

S4 Table Relative levels of HO1, NQO1, CYP1A1, CYP1B1 and AhR mRNAs. mRNA levels in Ishikawa cells exposed to different metals at their highest non-toxic concentrations for 24 and 48h (10 μM mercury, 3μM Cd, 300 μM Pb, 3 μM V) were measured by quantitative RT-PCR. A p-value < 0.05 was considered as statistically significant (*** p<0.001; ** p<0.01; * p<0.05) (n = 3).
(DOCX)

S5 Table Relative levels of HO1, NQO1, CYP1A1 and CYP1B1 mRNAs in Ishikawa cells exposed to either 10 μM HgCl_2_ or 25 nM TCDD alone or in combination for 48h. mRNAs levels were measured by quantitative RT-PCR. A p-value < 0.05 was considered as statistically significant (*** p<0.001; ** p<0.01; * p<0.05) (n = 3).
(DOCX)
