# Supplementary figures and images for: Determination of Heavy Metal Concentrations in Normal and Pathological Human Endometrial Biopsies and In Vitro Regulation of Gene Expression by Metals in the Ishikawa and Hec-1b Endometrial Cell Line
Source: PLoS One. 2015 Nov 23;10(11):e0142590. doi: 10.1371/journal.pone.0142590 (PMC4657954; doi:10.1371/journal.pone.0142590)

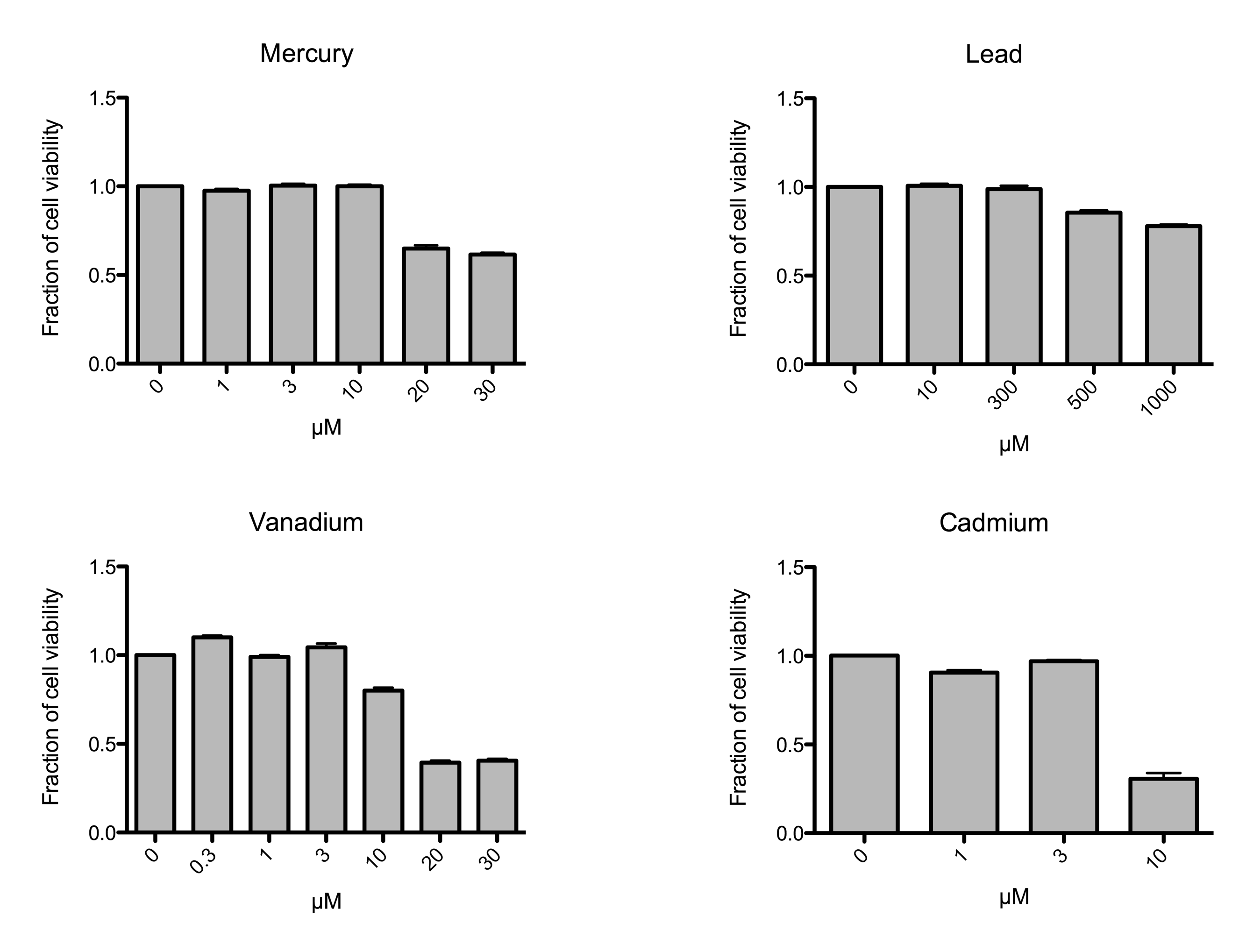

Supplement: S1 Fig — Ishikawa cells were treated with the various metals for 48h and the viability measured with the “CellTiter 96® Aqueous cell proliferation assay”. The results are expressed as the percentage of viability for untreated cells (100%) (n = 3). (TIF) [file pone.0142590.s001.tif]

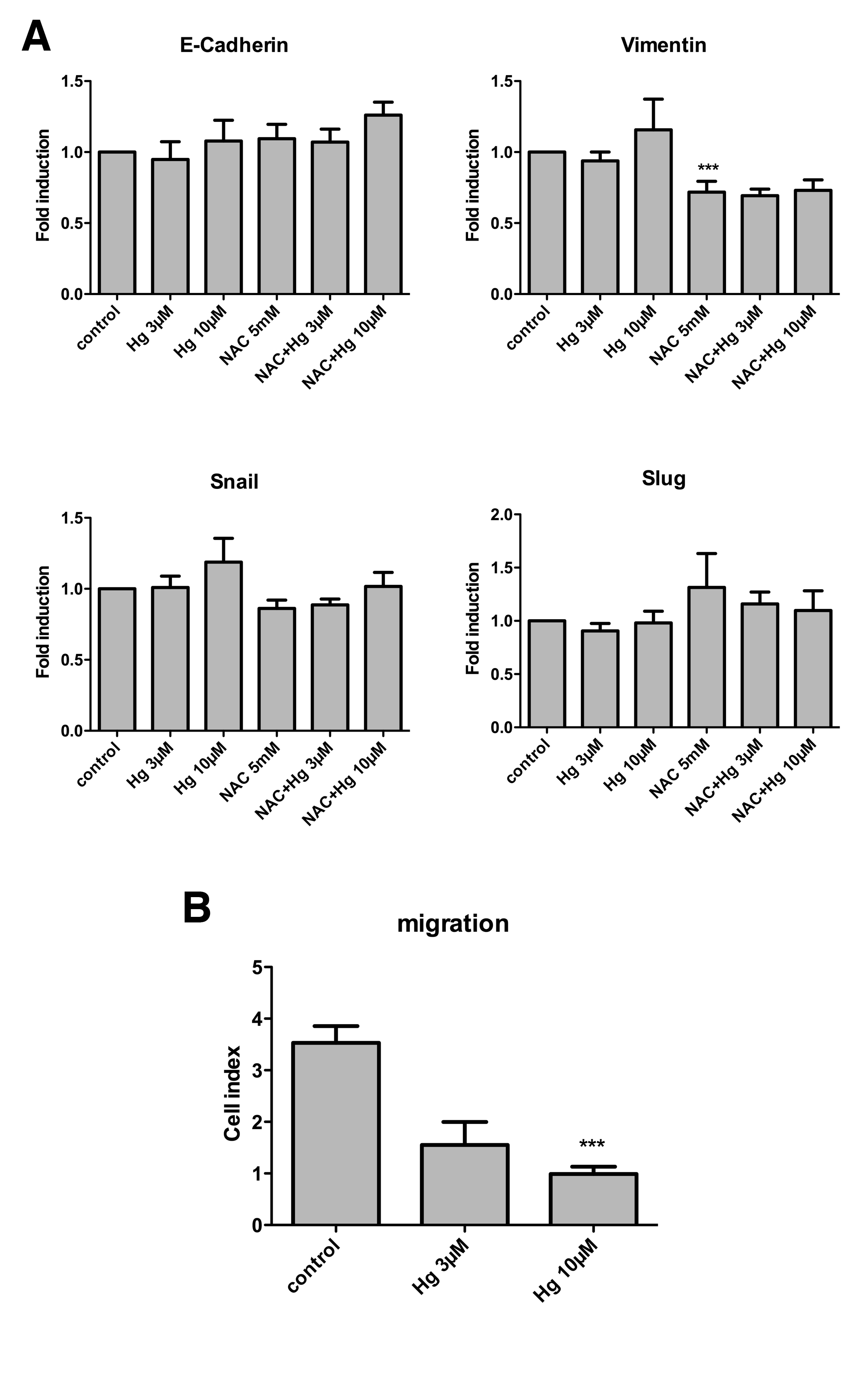

Supplement: S2 Fig — A. Relative mRNA levels of E-Cadherin, Vimentin, Snail and Slug in Ishikawa cells exposed or not for 48h to 3 or 10 μM HgCl2 or to 5 mM of N-AcetylCysteine (NAC) alone or in combination with mercury. Quantitative RT-PCR was used in this experiment. The results, from five independent experiments, are expressed as the mean ± SD (standard error of the mean). Differences between groups were analyzed by Student two-tailed t-tests. B. Effect of mercury on the rate of migration of Ishikawa cells. Ishikawa cells were incubated in the CIM -plate and treated with 3 or 10μM of mercury. The rate of migration was monitored in real-time using the xCELLigence system (n = 4). The results are expressed as measurements of the CI (***, P<0.001, n = 4). (TIF) [file pone.0142590.s002.tif]

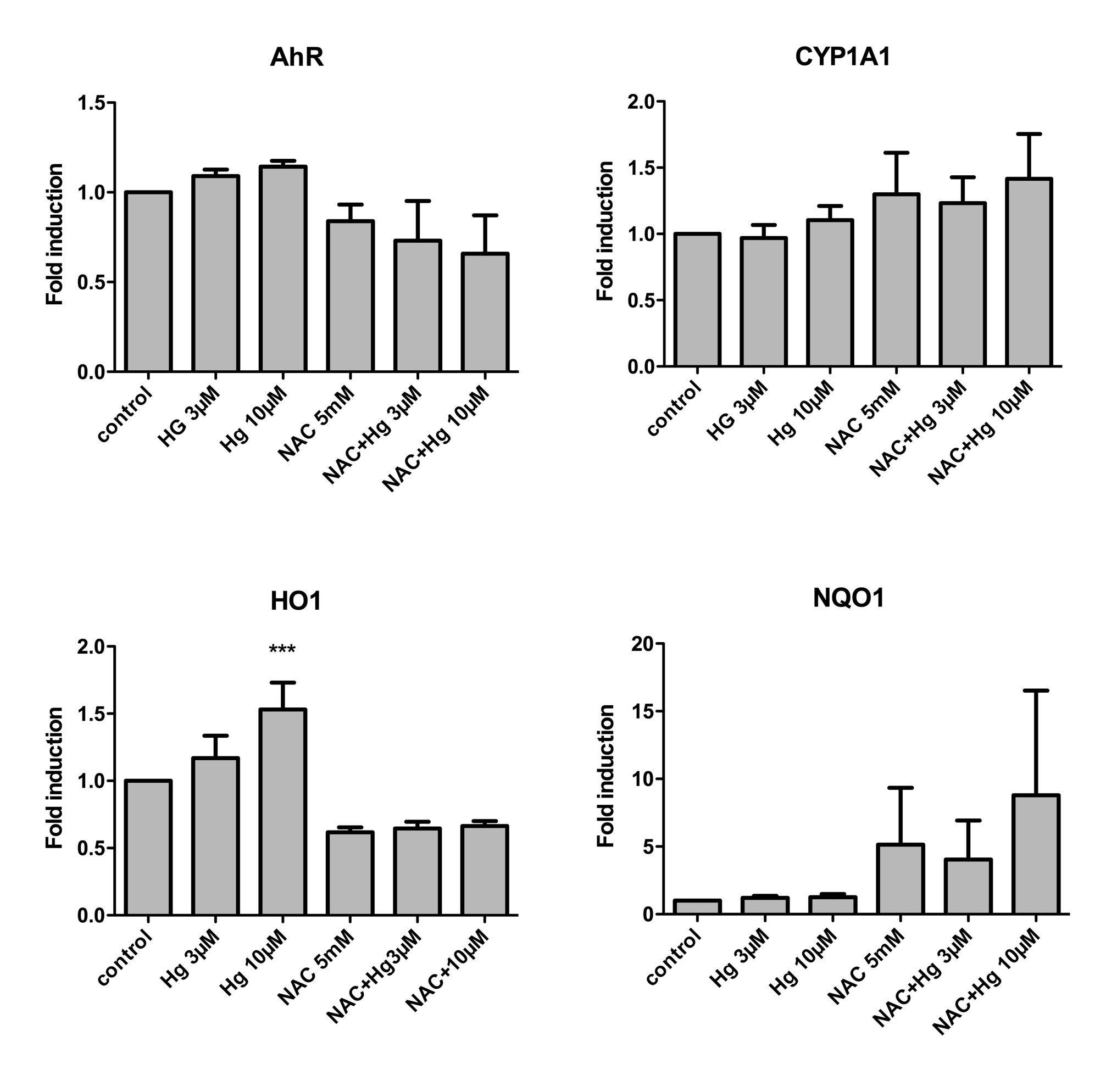

Supplement: S3 Fig — Quantitative RT-PCR was used in this experiment. The results, from five independent experiments, are expressed as the mean ± SD (standard error of the mean). Differences between groups were analyzed by Student two-tailed t-tests (***, P<0.001). (TIF) [file pone.0142590.s003.tif]

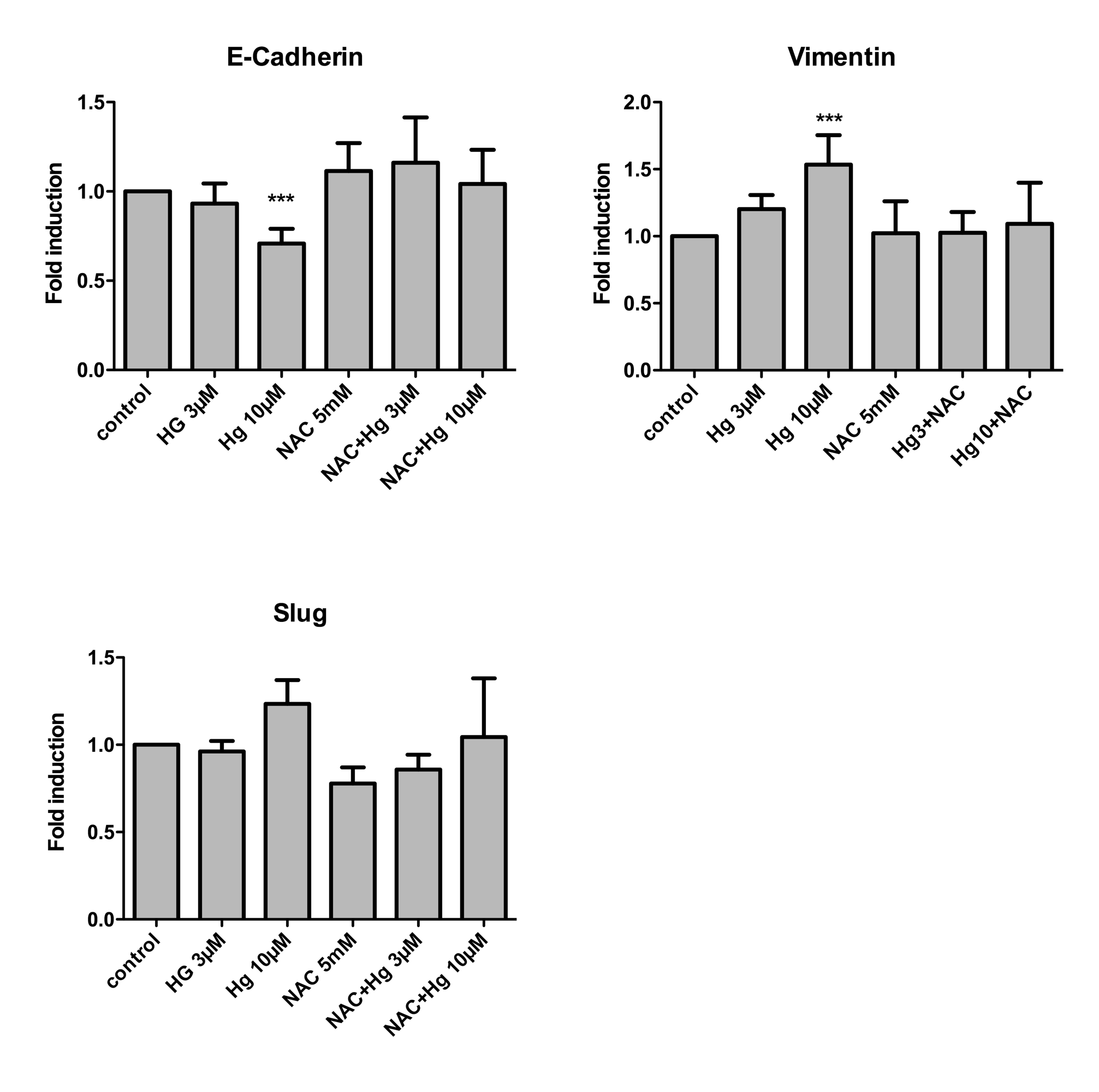

Supplement: S4 Fig — Quantitative RT-PCR was used in this experiment. The results, from five independent experiments, are expressed as the mean ± SD (standard error of the mean). Differences between groups were analyzed by Student two-tailed t-tests (***, P<0.001). (TIF) [file pone.0142590.s004.tif]
