# Supplementary material for: Determination of Heavy Metal Concentrations in Normal and Pathological Human Endometrial Biopsies and In Vitro Regulation of Gene Expression by Metals in the Ishikawa and Hec-1b Endometrial Cell Line
Source: PLoS One. 2015 Nov 23;10(11):e0142590. doi: 10.1371/journal.pone.0142590 (PMC4657954; doi:10.1371/journal.pone.0142590)
Supplement: S1 File — (DOCX) [file pone.0142590.s005.docx]

**Supplementary Material and Methods**

**Clinical studies**

**Tissue samples**

Human paraffin embedded samples were obtained from patients with different endometrial pathologies: typical endometrial hyperplasia, endometrial cancer and normal endometrial tissues. Atypical hyperplasias were excluded. Control samples were obtained from patients not having an identified endometrial pathology. All patients were pre-menopausal women less than 50 years old.

**Reagents and chemicals**

Suprapur^®^ Nitric acid 65% (Merck, Darmstadt, Germany) was used for microwave acid digestion.

**Biopsy preparation**

To remove the paraffin from the endometrial tissues, each sample was placed in a volume of toluene sufficient to cover the sample completely. After 30 minutes, the toluene was removed and replaced by absolute ethanol to dehydrate the sample and remove all traces of toluene. Each biopsy was weighed before and after drying. The dried sample weight represented 4.4 to 25.1% of that of the fresh sample. The dried samples were placed in 50mL microwave dedicated Teflon tubes. An appropriate volume of concentrated nitric acid was added to obtain a concentration of 10 g of dry tissue per liter. The Teflon tubes were tightened and placed into a microwave oven (DS-2000 Microwave Digestion System, CEM, ORSAY, France) and heated for 40 minutes. After complete digestion, an appropriate volume of MilliQ® water was added to obtain solutions containing 1g of dry tissue per liter. Metal-free control samples were also prepared and analyzed for each digestion series. The solutions were directly analyzed by inductively-coupled plasma mass spectroscopy (ICP-MS).

**Inductively-coupled plasma mass spectroscopy**

The detection and quantification of elements were performed using an ICP-MS Elan-DRCe apparatus (Perkin Elmer, Courtaboeuf, France). The signal intensity was measured as counts per second (cps). The ICP-MS operation parameters are described in S1 Table. A 1 mL.min^-1^ glass SeaSpray Concentric nebulizer (Perkin Elmer) and a 50mL cyclonic Baffled spray chamber from Perkin Elmer were used.

External calibrations for each element were performed using 4 solutions containing increasing amounts of Hg, Pb, Cd and V (0, 2, 5 and 10 µg/L). To matrix-match the samples, these solutions were prepared in a pre-digested bovine liver solution (1g tissue/L). In addition, the aqueous calibration was conducted using the same concentrations in order to probe the effect of the matrix on the measurement of the elements by ICP-MS. A slight effect of the matrix was noticed for Hg, Cd and V but the differences in the slopes between the calibrations for the aqueous and the liver samples did not exceed 13% (9.2% pour Hg). No effect was observed for Pb.

The limit of detection (LOD) was calculated for each element as 3 times the standard deviation of the apparent concentration of the blank. The relative standard deviation (RSD) was also calculated for each element. The LODs for Hg, Cd, Pb and V were respectively: 0.078 µg/L, 0.0014 µg/L, 0.023 µg/L and 1.16 µg/L.

**Statistical analysis**

The data are expressed as the mean ± SEM (standard error of the mean). Differences between groups were analyzed by Student two-tailed t-tests. Nemenyi’s test was used for the quantification of metals in biopsies. A p-value < 0.05 was considered to be statistically significant (*** p<0.001,** p<0.01; * p<0.05).

**Cell studies**

**Cell culture and chemical products**

The Ishikawa cell line is derived from an endometrial adenocarcinoma and was purchased from Sigma-Aldrich^TM^ (catalogue number 99040201). Ishikawa cells were cultured in Dulbecco's minimal essential medium (DMEM, Invitrogen, Cergy-Pontoise, France) supplemented with non essential amino acids and containing 10% fetal bovine serum (PAA), 200 U/mL penicillin (Invitrogen), 50 μg/mL streptomycin (Invitrogen) and 0.5 U/mL amphotericin B (Invitrogen) at 37 °C in a humidified 5% CO_2_ atmosphere. The HEC-1b cell line (AYCC number HTB-113) is derived from HEC-1A, which forms moderately well differentiated adenocarcinomas. They were cultured in the same conditions than Ishikawa cells. Metal salts (VoSO_4_ 5H_2_O), (HgCl_2_), (Pb(NO_3_)_2_), (CdCl_2_) were purchased from Sigma-Aldrich^TM^. Each metal salt was dissolved in water and sterile-filtered before cell treatment. Cells were incubated with various concentrations of metals or N-Acetyl Cysteine (10 mM) or TCDD (25 nM) for 24h or 48h.

**Reagents and chemicals**

Standard chloride solutions of Hg, Cd, Pb and V (Inorganic Ventures, distributed by Analab, Bischeim, France) were used to prepare working solutions by an appropriate dilution with MilliQ^®^ (Millipore, Molsheim, France) water containing 0.1M nitric acid or with a predigested bovine liver solution (1g tissue/L). N-Acetyl Cysteine (NAC) was obtained from Sigma. TCDD (99% in Nonane) was purchased from LCG Promochem (Molsheim, France).

**Cell counting - Cell viability assay**

The “CellTiter 96® Aqueous cell proliferation assay” (Promega^TM^, France) was used to determine the number of viable cells. Briefly, Ishikawa cells were plated into 96 well plates (5,000 cells per well) in 150 µL of culture medium. Forty-eight hours later, the medium was changed and the cells were treated with the metals for another 48h. Then, the medium was removed and replaced with 100 µL of fresh medium containing 20 µL of the reagent and the cells were kept at 37°C for 1h, according to the manufacturer’s instructions. The optical densities at 490 nm in each well were measured using a Microplate Power Wave X spectrophotometer (Biotek^TM^).

For cell counting, Ishikawa cells were plated into 12 well plates (100,000 cells per well) and treated with 3µM of HgCl_2_. Forty-eight hours later, the cells were trypsinized and counted using a Malassez counting chamber.

**RNA preparation and quantitative Reverse Transcription PCR**

For most experiments, 0.2 million cells were seeded into 6-well plates and treated, or not, 2 days later, with the compounds indicated in the Figures. RNA was prepared using the RNeasy mini kit from Qiagen (France). Reverse transcription was performed using the High Capacity cDNA Reverse Transcription Kit (Applied Biosystems, France) prior to quantitative PCR performed with 40 ng of cDNA (Bui et al. 2009), with duplicates for each experiment. The relative mRNA levels were measured using the ∆∆Ct method with RPL13A as the reference. The primers were: Cyp1A1 forward 5’-GGTCAAGGAGCACTACAAAACC-3’ and reverse 5’-TGGACATTGGCGTTCTCAT-3’, AhR forward 5’-TAACCCAGACCAGATTCCTC-34 and reverse 5’-GCAAACAAAGCCAACTGAG-3’, Cyp1B1 forward 5’-AACGTACCGGCCACTATCAC-3’ and reverse 5’-CAGTGGTGGCATGAGGAATA-3’, HO1 forward 5’-CGTTCCTGCTCAACATCC-3’ and reverse 5’-CTGTCGCACCAGAAAG-3’, NQO1 forward CAGCTCACCGAGAGAATAGT-3’ and reverse 5’-GAGTGAGCCAGTACGATCAGTG-3’, Vimentine forward 5’-CAGGAGGAGTGACTTCAGAG-3’ and reverse 5’-TGAGGTCAGGCTTGGAAAC-3’, E Cadherin forward 5’-GGACAGGGAGGATTTTGAGC-3’ and reverse 5’-GTGAAGGGAGATGTATTGGG-3’, Slug forward 5’-GGACCCACACATTACCTTG-3’ and reverse 5’-GAGCCCTCAGATTTGACC-3’, Snail forward 5’-TCCACAAGCACCAAGAGTC-3’ and 5’-CAGGCAGAGGACACAGAAC-3’ and RPL13A Forward AAGGTCGTGCGTCTGAAG-3’ and reverse 5’-GAGTCCGTGGGTCTTGAG-3’,

**Immunofluorescence**

Cells were seeded onto glass coverslips at a density of approximately 5x10^5^ cells per well in 6-well plates. Cells were treated with 3µM HgCl_2_ for 48 h in DMEM without phenol red and supplemented with 3% charcoal-stripped fetal calf serum. For immunofluorescence, all the steps were carried out at room temperature. The coverslips were washed twice in 1X PBS and then fixed in 4% paraformaldehyde for 20 min. The cells were permeabilized for 10 min in 0.3% PBS-Triton X100 and then incubated in PBS-1% bovine serum albumin, 0.3M glycine for 30 min. Incubations with the primary antibody paxillin (ab32084 from Abcam Paris, France) were performed for 1 h at room temperature in PBS-1% bovine serum albumin. For staining of actin and of the nucleus, FITC-conjugated phalloidin and TO-PRO-3 (Invitrogen) were included during the incubation with the secondary antibody. The coverslips were sealed with Dako Faramount Aqueous Mounting Medium Ready-to-use (Invitrogen). Mounted cells were observed and images recorded using a Zeiss LSM 510 confocal microscope (Carl Zeiss Meditec France SAS, Le Pecq, France) using a Zeiss 63x Plan-Apochromat O.N. = 1,4 / Oil / DIC objective and LSM Image Browser.

**xCELLigence real-time cell activity**

Endometrial cells were seeded in duplicate for each condition into 16-well xCELLigence E-plates at 10,000 cells/well in a final volume of 160

μL and immediately treated or not with 3 or 10µM HgCl_2_. The impedance value of each well was automatically monitored by the xCELLigence system and expressed as a cell index value (CI). Initial attachment and spreading were monitored by measuring the CI every 15 minutes for the first twenty hours. The rate of attachment (CI/h) was calculated using the xCELLigence RTCA software (ACEA Biosciences, Inc., Roche Diagnostics). Each experiment was performed in triplicate. For migration studies, Ishikawa cells (40,000 cells per well) were seeded into CIM-16 plates (which are specifically used to measure cell migration) in DMEM without phenol red supplemented with 3% charcoal-treated (desteroidized) FBS. The lower chambers contained media with 10% FBS in order to assess chemotactic migration. After 24 h of incubation at 37°C, the medium was replaced and the cells were treated, or not, with 3 or 10 µM of HgCl_2_. The impedance value of each well was automatically monitored by the xCELLigence system and expressed as a cell index value (CI). Higher CI values equate to more migration; the cells migrate from the upper chamber towards the lower one. The migration curves were monitored online every 5 min during the incubation and the slope of the migration curve was calculated using the RTCA 1.2 software. Each experiment was performed in triplicate (n=4).

**Statistical analysis**

For the *in vitro* studies, the values are expressed as mean ± SD. The data were analyzed by analysis of variance (ANOVA) followed by Fisher's Least Significant Difference test to examine the differences between the different groups. A value of p < 0.05 was considered to be statistically significant.
