# Supplementary material for: Determination of Heavy Metal Concentrations in Normal and Pathological Human Endometrial Biopsies and In Vitro Regulation of Gene Expression by Metals in the Ishikawa and Hec-1b Endometrial Cell Line
Source: PLoS One. 2015 Nov 23;10(11):e0142590. doi: 10.1371/journal.pone.0142590 (PMC4657954; doi:10.1371/journal.pone.0142590)
Supplement: S1 Table — (DOCX) [file pone.0142590.s006.docx]

**Supplementary Table 1: ICP-MS parameters**

| Nebulizer | Glass SeaSpray Concentric (nominal flow: 1 mL.min^-1^) |
| --- | --- |
| **Spray chamber** | Glass cyclonic baffled (50 mL) |
| **Nebulizer gas flow** | 0.92 mL min^-1^ |
| **Auxiliary gas flow** | 1.20 mL min^-1^ |
| **Plasma gas flow** | 15 L min^-1^ |
| **ICP RF power** | 1125 W |
| **O_2_ flow rate (DRC mode)** | 0.60 (0.1 – 1 mL.min^-1^) |
| **RPQ** | 0.25 (0.05 – 0.7) |
| **Pulse stage voltage** | 800 V |
| **Analog stage voltage** | -1612.5 V |
| **Measured *m/z*** | ^202^Hg, ^208^Pb, ^114^Cd and ^51^V |
| **Scan mode** | Peak hopping |
| **Dwell time per isotope** | 250 ms |
