# Supplementary material for: Determination of Heavy Metal Concentrations in Normal and Pathological Human Endometrial Biopsies and In Vitro Regulation of Gene Expression by Metals in the Ishikawa and Hec-1b Endometrial Cell Line
Source: PLoS One. 2015 Nov 23;10(11):e0142590. doi: 10.1371/journal.pone.0142590 (PMC4657954; doi:10.1371/journal.pone.0142590)
Supplement: S2 Table — A p-value < 0.05 was considered as statistically significant (*** p<0.001; ** p<0.01; * p<0.05) (n = 3). (DOCX) [file pone.0142590.s007.docx]

| **Gene** | **HO1** | | **NQO1** | | **CYP1A1** | **CYP1B1** | **AhR** | |
| --- | --- | --- | --- | --- | --- | --- | --- | --- |
| **HgCl_2_ (µM) – 24h** | **Fold induction of mRNA level over the control** | | | | | | | |
| 0.30 | **1.90 | 1.32 | | 1.09 | | 1.00 | | 1.01 |
| 1.00 | **1.43 | 1.19 | | 1.23 | | 1.16 | | 1.38 |
| 3.00 | **2.38 | *1.62 | | 1.43 | | 1.05 | | 1.46 |
| 10.00 | **5.39 | *1.61 | | 1.39 | | 1.02 | | 1.28 |
| 20.00 | **48.57 | *1.70 | | **3.78 | | 1.37 | | 1.90 |

**Supplementary Table 2:** Relative levels of HO1, NQO1, CYP1A1, CYP1B1 and AhR mRNA in Ishikawa cells exposed to different Hg concentrations for 24h measured by quantitative RT-PCR. A p-value < 0.05 was considered as statistically significant (*** p<0.001; ** p<0.01; * p<0.05) (n=3).
