# Supplementary material for: Determination of Heavy Metal Concentrations in Normal and Pathological Human Endometrial Biopsies and In Vitro Regulation of Gene Expression by Metals in the Ishikawa and Hec-1b Endometrial Cell Line
Source: PLoS One. 2015 Nov 23;10(11):e0142590. doi: 10.1371/journal.pone.0142590 (PMC4657954; doi:10.1371/journal.pone.0142590)
Supplement: S3 Table — A p-value < 0.05 was considered as statistically significant (*** p<0.001; ** p<0.01; * p<0.05). UD: undetermined (n = 3). (DOCX) [file pone.0142590.s008.docx]

| **Gene** | **HO1** | **NQO1** | **CYP1A1** | **CYP1B1** | **AhR** |
| --- | --- | --- | --- | --- | --- |
| **HgCl_2_ (µM) – 48h** | **Fold induction of mRNA level over the control** | | | | |
| 0.00 | 1.00 | 1.00 | 1.00 | 1.00 | 1.00 |
| 0.30 | *1.39 | *1.96 | 0.72 | 1.03 | 1.28 |
| 1.00 | 1.03 | *1.55 | 1.17 | 1.23 | 1.52 |
| 3.00 | *1.67 | ***2.45 | 1.14 | 1.02 | 1.55 |
| 10.00 | *4.07 | **3.34 | 1.14 | 1.17 | 1.05 |
| 20.00 | **19.39 | ***4.42 | **2.89 | UD | 1.93 |

**Supplementary Table 3:** Relative levels of HO1, NQO1, CYP1A1, CYP1B1 and AhR mRNAs in Ishikawa cells exposed to different Hg concentrations for 48h measured by quantitative RT-PCR. A p-value < 0.05 was considered as statistically significant (*** p<0.001; ** p<0.01; * p<0.05). UD: undetermined (n=3).
