# Supplementary material for: Determination of Heavy Metal Concentrations in Normal and Pathological Human Endometrial Biopsies and In Vitro Regulation of Gene Expression by Metals in the Ishikawa and Hec-1b Endometrial Cell Line
Source: PLoS One. 2015 Nov 23;10(11):e0142590. doi: 10.1371/journal.pone.0142590 (PMC4657954; doi:10.1371/journal.pone.0142590)
Supplement: S4 Table — mRNA levels in Ishikawa cells exposed to different metals at their highest non-toxic concentrations for 24 and 48h (10 μM mercury, 3μM Cd, 300 μM Pb, 3 μM V) were measured by quantitative RT-PCR. A p-value < 0.05 was considered as statistically significant (*** p<0.001; ** p<0.01; * p<0.05) (n = 3). (DOCX) [file pone.0142590.s009.docx]

**A**

| 24 h | Hg | Cd | Pb | V |
| --- | --- | --- | --- | --- |
| HO1 | *5.39 | ***21.58 | ***4.07 | 0.96 |
| NQO1 | ***1.61 | *1.54 | ***2.04 | 0.94 |
| CYP1A1 | 1.39 | **2.39 | 1.58 | 0.93 |
| CYP1B1 | 1.02 | 1.21 | 1.51 | 1.17 |

**B**

| 48 h | Hg | Cd | Pb | V |
| --- | --- | --- | --- | --- |
| HO1 | *4.07 | **13.21 | ***5.23 | 1.29 |
| NQO1 | **3.34 | ***1.73 | ***3.67 | 0.99 |
| CYP1A1 | 1.14 | *1.80 | 1.50 | 1.16 |
| CYP1B1 | 1.17 | 1.12 | 1.33 | 1.09 |

**Supplementary Tables 4A and 4B:** Relative levels of HO1, NQO1, CYP1A1, CYP1B1 and AhR mRNAs in Ishikawa cells exposed to different metals at their highest non-toxic concentrations for 24 and 48h (10 µM Hg, 3µM Cd, 300 µM Pb, 3 µM V) measured by quantitative RT-PCR. A p-value < 0.05 was considered as statistically significant (*** p<0.001; ** p<0.01; * p<0.05) (n=3).
