# Supplementary material for: Determination of Heavy Metal Concentrations in Normal and Pathological Human Endometrial Biopsies and In Vitro Regulation of Gene Expression by Metals in the Ishikawa and Hec-1b Endometrial Cell Line
Source: PLoS One. 2015 Nov 23;10(11):e0142590. doi: 10.1371/journal.pone.0142590 (PMC4657954; doi:10.1371/journal.pone.0142590)
Supplement: S5 Table — mRNAs levels were measured by quantitative RT-PCR. A p-value < 0.05 was considered as statistically significant (*** p<0.001; ** p<0.01; * p<0.05) (n = 3). (DOCX) [file pone.0142590.s010.docx]

| Gene | **HO1** | **NQO1** | **CYP1A1** | **CYP1B1** |
| --- | --- | --- | --- | --- |
| Treatment (48h) | **Fold increase over control** | | | |
| Hg 10 µM | 4.07* | 3.34** | 1.14 | 1.16 |
| TCDD 25 nM | 1.60 | 1.44 | 201*** | 22.2*** |
| Hg + TCDD | 4.14* | 4.21** | 284*** | 37*** |

**Supplementary Table 5:** Relative levels of HO1, NQO1, CYP1A1 and CYP1B1 mRNAs in Ishikawa cells exposed to either 10 µM Hg or 25 nM TCDD alone or in combination for 48h measured by quantitative RT-PCR. A p-value < 0.05 was considered as statistically significant (*** p<0.001; ** p<0.01; * p<0.05) (n=3).
